# High Expression of Cannabinoid Receptor 2 on Cytokine-Induced Killer Cells and Multiple Myeloma Cells

**DOI:** 10.3390/ijms21113800

**Published:** 2020-05-27

**Authors:** Francesca Garofano, Ingo G. H. Schmidt-Wolf

**Affiliations:** Department of Integrated Oncology, Center for Integrated Oncology (CIO), University Hospital Bonn, 53127 Bonn, Germany; Francesca.Garofano@ukbonn.de

**Keywords:** cannabidiol, adoptive cellular immunotherapy, multiple myeloma, cytokine-induced killer cells, endocannabinoid system

## Abstract

Multiple myeloma (MM) is characterized by aberrant bone marrow plasma cell (PC) proliferation and is one of the most common hematological malignancies. The potential effect of cannabinoids on the immune system and hematological malignancies has been poorly characterized. Cannabidiol (CBD) may be used to treat various diseases. CBD is known to exert immunomodulatory effects through the activation of cannabinoid receptor 2 (CB2), which is expressed in high levels in the hematopoietic system. Cytokine-induced killer (CIK) cells are a heterogeneous population of polyclonal T lymphocytes obtained via ex vivo sequential incubation of peripheral blood mononuclear cells (PBMCs) with interferon-γ (IFN-γ), anti CD3 monoclonal antibody, and IL-2. They are characterized by the expression of CD3+ and CD56+, which are surface markers common to T lymphocytes and natural killer (NK) cells. CIK cells are mainly used in hematological patients who suffer relapse after allogeneic transplantation. Here, we investigated their antitumor effect in combination with pure cannabidiol in KMS-12 MM cells by lactate dehydrogenase LDH cytotoxicity assay, CCK-8 assay, and flow cytometry analysis. The surface and intracellular CB2 expressions on CIK cells and on KMS-12 and U-266 MM cell lines were also detected by flow cytometry. Our findings confirm that the CB2 receptor is highly expressed on CIK cells as well as on MM cells. CBD was able to decrease the viability of tumor cells and can have a protective role for CIK cells. It also inhibits the cytotoxic activity of CIKs against MM at high concentrations, so in view of a clinical perspective, it has to be considered that the lower concentration of 1 µM can be used in combination with CIK cells. Further studies will be required to address the mechanism of CBD modulation of CIK cells in more detail.

## 1. Introduction

The human endocannabinoid system (ECS), which includes the cannabinoid receptors, endocannabinoids, and their metabolizing enzymes, has been considered as a pharmacological target for the treatment of various cancer types including multiple myeloma. Two major cannabinoids extracted from *Cannabis sativa* plant, called phytocannabinoids, (-)delta9-tetrahydrocannabinol (THC) [1] and cannabidiol (CBD) [2] which bind to cannabinoid receptors, are the most researched compounds and have been recently used for the treatment of cancer not only for their palliative effects like the treatment of pain and inhibition of vomiting associated with chemotherapy but also as antitumor drugs based on their potential antitumor activity. The best studied two endogenous cannabinoid receptors are cannabinoid receptor CB1 and CB2. They are members of the G-protein coupled receptors (GPCR) family and can signal through G-proteins of the G _i/0_ type [3]. The CB1 (encoded by the CNR1 gene) is expressed at high levels in the brain and at low levels in the hematopoietic system. Instead, the CB2 [4] (encoded by the CNR2 gene) is predominantly expressed in cells and tissues of the immune system and at low levels in the non-hematopoietic cells in the brain. The gene for CB2 receptor is located on chromosome 1p36 in humans and contains a single coding exon which is encoding for a 360-amino-acid-long single polypeptide chain. This comprises seven transmembrane alpha-helices with an extracellular glycosylated N-terminus and an intracellular C-terminus which is involved in signal transduction [5]. After engagement with an agonist ligand, CB2 is internalized and desensitized and an inverse agonist is able to reverse this process. CBD is an antagonist of CB1 and CB2 receptor agonists [6], and it also acts as an inverse agonist at CB1 and CB2. CBD can also interact with other molecular targets like vanilloid receptors (e.g., the transient receptor potential vanilloid type-1 and 2 TRPV1-2) [7], G protein-coupled receptor 55 (GPR55) [8], and peroxisome proliferator-activated receptor gamma (PPARgamma) [9]. It is of particular interest since it is not psychoactive but has significant relaxing, anti-inflammatory, pain-relieving, and immunomodulatory properties [10,11]. Current research supports the concept that CB2 is a promising therapeutic target for immune modulation. Although both hematopoietic and immune systems express high levels of CB2, the effect of cannabinoids on the immune system and hematological malignancies are poorly characterized. CB2 is active constitutively only on specific cells populations [12] and is expressed in high levels in B-cells which are precursors of plasma cells (PCs). Multiple myeloma (MM) is a PC malignancy and is one of the most common hematological malignancies. It is characterized by aberrant bone marrow PCs proliferation with excessive monoclonal protein production. It has been demonstrated that cannabinoids can induce a selective apoptosis in MM cell lines and PCs of MM patients which was mediated by caspase activation, mainly caspase-2, without harming normal cells and that blockage of the CB2 inhibited cannabinoid-induced apoptosis [13]. Morelli MB et al. reported that CBD strongly inhibited growth, arrested cell cycle progression, and induced MM cells death by regulating the ERK, AKT, and NF-κB pathways with major effects in TRPV2 positive cells [14,15]. It has been previously shown that CB2 signaling in unstimulated human immunocompetent primary leukocytes (peripheral blood mononuclear cells (PBMCs)) induced the secretion of IL-6 and IL-10 [16].

The use of immune therapy for the treatment of hematological malignancies is an effective obvious treatment for hematological malignancies, as they are more accessible to effector immune cells. Cytokine-induced killer (CIK) cells are easily developed ex vivo from PBMCs by adding the interferon-γ (IFN-γ), anti-CD3 mAb, IL-2, and IL-1β. CIK cells express the T-cell receptor CD3 as well as the natural killer cell receptor NKG2D (natural killer group 2 member D) that is thought to be responsible for the specific targeting of tumor cells [17,18]. The addition of IFN-γ during generation of CIK cells activates monocytes, providing them with a contact-dependent factor CD58 (lymphocyte function associated antigen-3 (LFA-3)) and a soluble factor IL-12. These two factors are important for the expansion to CD56-positive T cells and the acquisition of T helper 1 phenotype of CIK cells. The addition of anti-CD3 acts as a mitogenic stimulus and high doses of IL-2 principally promote the expression of the natural killer group 2 member D (NKG2D) transmembrane adapter protein DAP10, which is essential for cytolysis. CIK cells are able to secrete TNF-α, IL-2, and IL-6. The mechanism of CIK-associated tumor cytotoxicity has not been fully elucidated yet, including the efficiency of combining CIK and cannabidiol on tumor cells.

## 2. Results

### 2.1. CB2 is Detectable by Flow Cytometry on Days 7 and 14 in CIK Cells

**Figure 1:**
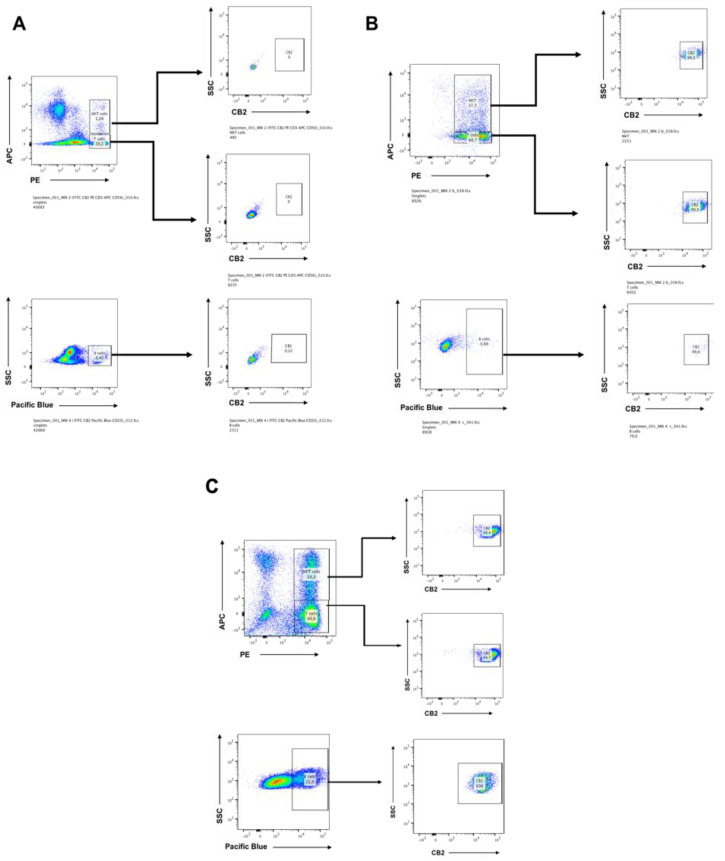
(**A**) The differential expression of CB2 receptor in the main cell subsets of human Cytokine-induced killer (CIK) cells, i.e., CD3+ T lymphocytes (CD4+ and CD8+), CD3+CD56+ NKT cells, and CD20+ B-cells. Both surface expression and intracellular CB2 expression were evaluated. The CIK cells were immunophenotyped at day 0 by flow cytometry. At day 0, CB2 expression was not detectable in any T lymphocytes subsets (CD3+ T lymphocytes 20.0%, CD3+CD56+ NKT 1.2%). Also, the B cells subset which was 5.4% did not express any CB2 receptor. (**B**) The CIK cells were immunophenotyped at day 14 by flow cytometry. Both surface expression and intracellular CB2 expression were evaluated. The T lymphocytes subsets were CD3+ T lymphocytes 66.7% and CD3+CD56+ NKT 27.3%. CB2 expression was dramatically upregulated within both CD3+ T lymphocytes (CD4+ and CD8+) 99.3% and the CD3+CD56+ NKT cells population 98.1%. The B cells subset was only the 0.9%. (**C**) The CIK cells were immunophenotyped on day 7 by flow cytometry. Both surface expression and intracellular CB2 expression were evaluated. The T lymphocytes subsets were CD3+ T lymphocytes 60.8% and CD3+CD56+ NKT 18.3%. CB2 expression was upregulated within both CD3+ T lymphocytes (CD4+ and CD8+) 99.7% and the CD3+CD56+ NKT cells population 99.4%. The B cells subset was the 21%, and all were positive for CB2 100%.

Initial studies were performed to assess the differential expression of CB2 receptor in the main cell subsets of human CIK cells, i.e., CD3+ T lymphocytes (CD4+ and CD8+), CD56+ NK cells. We also analyzed CD20+ B cells and evaluated for both surface expression and intracellular CB2 expression by flow cytometry. The CIK cells were immunophenotyped at day 0 by flow cytometry. At day 0, CB2 expression was not detectable in any T lymphocytes subsets and B cells (Figure 1A). When CIK cells were activated at day 14, the CB2 expression was dramatically upregulated within the natural killer T (NKT) population CD3+CD56+T cells (Figure 1B) 98.1%. We also immunophenotyped CIK cells on day 7 in order to see at early stage the expression of CB2 receptor in the different populations (Figure 1C). 

### 2.2. LDH Release of CIK Cells Significantly Decreases with CBD

**Figure 2:**
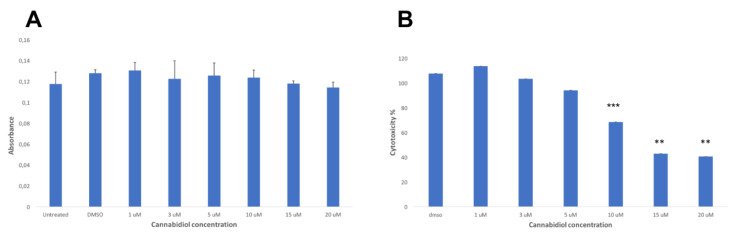
(**A**) The cell viability in CIKs exposed to various concentrations of Cannabidiol (CBD) for 24 h: The results of the CCK8 assay did not show any significant difference compared to the DMSO control (*p* > 0.05). (**B**) The cytotoxicity in CIKs exposed to various concentrations of CBD for 24 h: The results of the LDH assay showed a significant decrease of the LDH release at 10 µM (*p* ≤ 0.001), 15 µM, and 20 µM (*p* ≤ 0.01) compared to the DMSO control.

The CCK8 assay was used as a direct measure of cell viability in CIKs exposed to various concentrations of CBD from 1 µM to 20 µM for 24 h. The results did not show any significant difference compared to the dimethyl sulfoxide (DMSO) control (Figure 2A). However, when the LDH assay was used as a direct measure of cytotoxicity in CIKs exposed to various concentrations of CBD from 1 µM to 20 µM for 24 h, the results showed that the LDH release of CIK cells was significantly decreased compared to the DMSO control (Figure 2B).

### 2.3. CB2 is Highly Expressed in Multiple Myeloma Cell Lines

**Figure 3:**
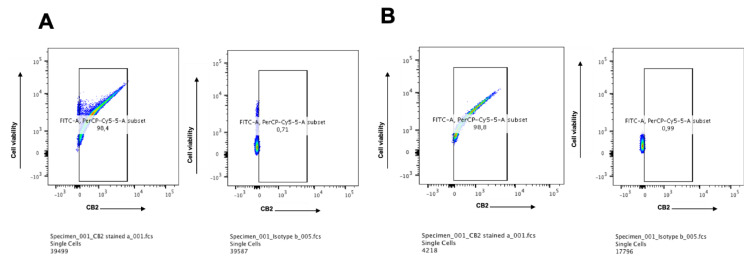
(**A**) Protein expression of the CB2 receptor on KMS-12 PE multiple myeloma cell line: Both intracellular and surface expressions of CB2 were detected by flow cytometry. The results showed that 98.4% of the cells express CB2 while the corresponding isotype control was 0.7%. (**B**) Protein expression of the CB2 receptor on U-266 multiple myeloma cell line: Both intracellular and surface expressions of CB2 were detected by flow cytometry. The results showed that 98.8% of the cells express CB2 while the corresponding isotype control was 1.0%.

Both surface and intracellular expressions of CB2 in KMS-12 PE and U-266 multiple myeloma cell lines were detected via flow cytometry analysis. The KMS-12 PE cell line which was established from the pleural effusion showed a very high level of CB2 receptor expression 98.4% (Figure 3A), and the U-266 cell line which was reported to produce IL-6 showed a very high level 98.8% (Figure 3B). This is in accordance with other studies showing the distribution of CB2 in the cells of the immune system where B lymphocytes express the highest amounts of CB2 followed in order by NK cells, macrophages, and T lymphocytes.

### 2.4. CBD Significantly Increases the LDH Release of KMS Myeloma Cells at High Concentrations

**Figure 4:**
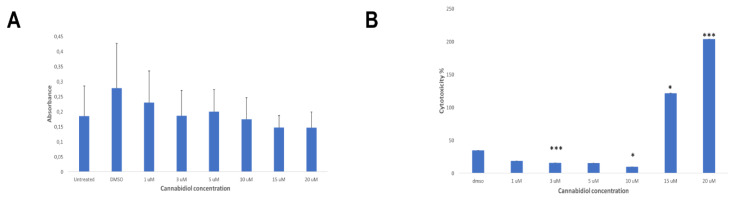
(**A**) The cell viability in KMS-12 PE cells exposed to various concentrations of CBD for 24 h: The results of the CCK8 assay did not show any significant difference compared to the DMSO control (*p* > 0.5). (**B**) The cytotoxicity in in KMS-12 PE cells exposed to various concentrations of CBD for 24 h: The results of the LDH assay showed a significant decrease of the LDH release at 3 µM (*p* ≤ 0.001) and 10 µM (*p* < 0.05) compared to the DMSO control. However, the results showed a significant increase at 15 µM (*p* < 0.05) and 20 µM (*p* ≤ 0.001) compared to the DMSO control.

The CCK8 assay was used as a direct measure of cell viability in KMS-12 PE cells exposed to various concentrations of CBD from 1 µM to 20 µM for 24 h. The results did not show any significant difference compared to the DMSO control (Figure 4A). However, when the LDH assay was used as a direct measure of cytotoxicity in KMS-12 PE cells exposed to various concentrations of CBD from 1 µM to 20 µM for 24 h, the results showed that the LDH release of KMS-12 PE cells was significantly increased compared to the DMSO control (Figure 4B).

### 2.5. CBD Significantly Decreases the Cytotoxicity of CIK Cells Against KMS Cells at High Concentrations

**Figure 5:**
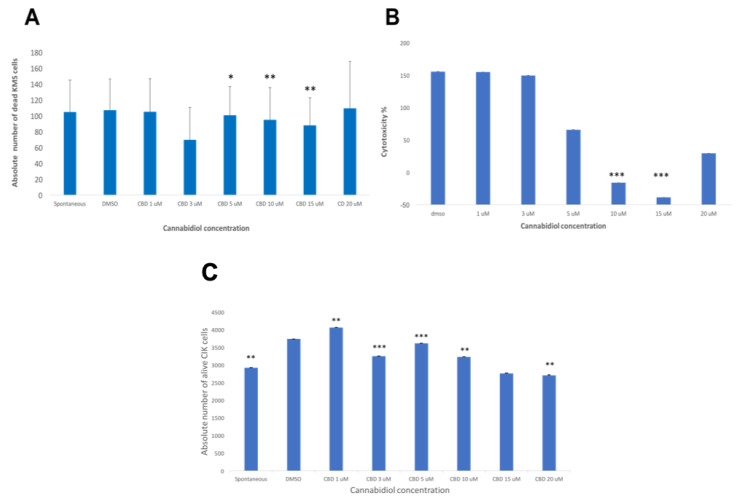
(**A**) The absolute number of dead KMS-12 PE cells was calculated for each sample condition of co-cultured CIKs and KMS-12 PE cells exposed to various concentrations of CBD for 24 h via flow cytometry analysis. The results showed a significant decrease of the absolute number of the dead KMS-12 PE cells at 5 µM (*p* < 0.05), 10 µM, and 15 µM (*p* ≤ 0.01) compared to the DMSO control. (**B**) The cytotoxicity of the effector CIKs against target KMS-12 PE cells exposed to various concentrations of CBD for 24 h was analyzed via LDH assay. The results of the LDH assay showed a significant decrease of the LDH release at 10 µM and 15 µM (*p* ≤ 0.001) compared to the DMSO control. (**C**) The absolute number of alive CIK cells (CD3+CD56+ NKT cells population) was calculated for each sample condition of co-cultured CIKs and KMS-12 PE cells exposed to various concentrations of CBD for 24 h via flow cytometry analysis. The results showed a significant decrease of the absolute number of the alive CIK cells compared to the DMSO control (*p* ≤ 0.01) and a significant increase of the absolute number of the alive CIK cells at 1 µM (*p* < 0.001), 3 µM (*p* ≤ 0.001), 5 µM (*p* ≤ 0.001), and 10 µM (*p* ≤ 0.01) compared to the DMSO control. There was a significant decrease at 20 µM (*p* ≤ 0.01).

When finally the effector cells were co-cultured with the target cells (KMS-12 PE) with an E:T ratio 10:1 and exposed to various concentrations of CBD from 1 µM to 20 µM for 24 h, a concentration-dependent inhibition response was recorded via flow cytometry analysis (Figure 5A). Similar results were obtained when the LDH cytotoxicity experiment was used (Figure 5B). We also reported the absolute number of viable residual CIK cells (CD3+CD56+ NKT cells population) after treatment with CBD from 1 µM to 20 µM (Figure 5C). The results confirmed that CBD has a protective role for CIK cells at a concentration of 1 µM.

### 2.6. CBD Inhibits the Growth of NKT Cells

**Figure 6:**
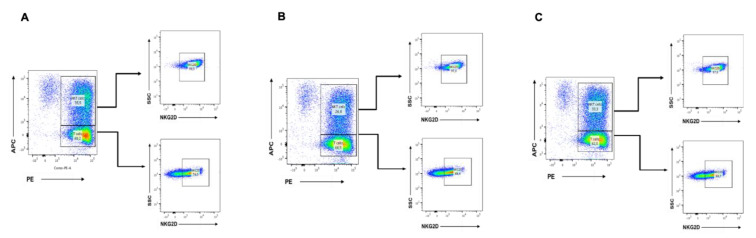
(**A**) The differential expression of the natural killer group 2 member D (NKG2D) receptor in the main cell subsets of human CIK cells, i.e., CD3+ T lymphocytes (CD4+ and CD8+) and CD3+CD56+ NKT cells: The surface NKG2D expression was evaluated. The CIK cells were immunophenotyped at day 14 by flow cytometry. In the untreated, both T lymphocytes subsets CD3+ T lymphocytes 60.2% and CD3+CD56+ NKT 35.5% showed high expressions of NKG2D receptor 74% and 98.5%, respectively. (**B**) In the untreated CBD 10 µM, both T lymphocytes subsets CD3+ T lymphocytes 68.5% and CD3+CD56+ NKT 26.8% showed high expressions of NKG2D receptor 68.4% and 97%, respectively. (**C**) In the DMSO control, both T lymphocytes subsets CD3+ T lymphocytes 62% and CD3+CD56+ NKT 33.3% showed high expressions of NKG2D receptor 69.7% and 97.8%, respectively.

We performed some studies to assess the differential expression of NKG2D receptor in the main cell subsets of human CIK cells, i.e., CD3+ T lymphocytes (CD4+ and CD8+) and CD56+ NK cells for surface NKG2D expression by flow cytometry. CIK cells were differentiated either with DMSO alone, CBD 10 µM, or untreated. CIK cells were immunophenotyped at day 14 by flow cytometry. The results showed that CBD 10 µM was able to decrease the percentage of NKT cells compared to the untreated and DMSO control (Figure 6). However, the percentage of NKG2D-positive NKT cells did not change between them. 

## 3. Discussion

Endocannabinoids have been reported to affect immune function, and the cannabinoid receptor that, for the most part, is linked to the modulation of the immune responses is CB2. The factors and conditions that regulate CB2 expression are still poorly understood, and the signaling cascades are incomplete. Cannabinoids also have been reported to suppress a variety of activities of T lymphocytes in a mode that appears to be linked functionally to CB2. The results from a number of studies suggested that cannabinoids not only exert direct effects on immune cells but also elicit a shift in the cytokine expression profile from that which is pro-inflammatory (Th1) to one that is anti-inflammatory (Th2). Cytokine-induced killer (CIK) cells are an heterogenous T cell population capable of exerting a potent major histocompatibility complex (MHC)-unrestricted cytotoxicity against both hematologic and solid tumors but not hematopoietic precursors and normal tissues [19]. Within the heterogeneous T cell population, two main subpopulations can be distinguished, one coexpressing the CD3 and CD56 molecules (range: 40% to 80%) while the other presenting a CD3+ CD56- phenotype (range: 20% to 60%). It also comprises a small subpopulation (<10%) of CD3- CD56+ NK cells [20]. The antitumor efficacy of CIKs has been reported to be associated with the CD3+CD56+ subset that serves as the main effector cells combining T cell capability with NK cell function. It has been demonstrated that cannabinoids can induce a selective apoptosis in MM cell lines and PCs of MM patients which was mediated by caspase activation and that CIK cells were resistant to Fas-mediated apoptosis that could be induced by the expression of FasL on cancer cells.

Although the expression and function of CB1 and CB2 receptors has been widely studied in several immune cells, as yet, no studies have addressed the expression of CB2 on CIK cells. In the attempt to shed some light on the CIK cells of subsets that could express CB2, we performed polychromatic flow cytometry that allowed simultaneous interrogation of receptor expression in distinct cell populations. Such a multiparametric approach revealed remarkable high levels of CB2 expression in KSM-12 PE and U-266 cell lines in CIK cells on days 7 and 14. Here, we demonstrate that cannabinoid exerts an inhibition of CIK cytotoxic function against multiple myeloma (MM) cell line KMS-12 PE at high concentrations. Additionally, this is the first study reporting a detailed characterization of CB2 expression in human mature CIK cells and these two tumor cell lines by using flow cytometry directly.

The results of the LDH cytotoxicity assay showed that CBD can affect the viability of MM cells. These results confirm the antitumor potential of CBD against MM, which was already reported by other studies based on the analysis of apoptosis. However, CBD can be protective regarding CIK cells since the LDH release was decreasing in a dose-dependent manner. In view of a clinical perspective, it has to be considered that the lower concentration of 1 µM could be used in the combination with CIK cells. Further studies will be required to address the mechanisms of the CIK cells whereby CB2 modulates interaction in more detail. 

## 4. Materials and Methods

### 4.1. Generation of Cytokine-Induced Killer Cells

Peripheral blood mononuclear cells (PBMC) were isolated from blood samples of healthy donors after obtaining written informed consents, and isolation was carried out on the same day or kept overnight at 4 °C for use on the next day. Blood was mixed with Dulbecco’s phosphate-buffered saline (DPBS; PAN BIOTECH, Aidenbach, Germany)-ethylenediaminetetracetic acid (EDTA; Life Technologies, PAA, Cölbe, Germany) (1:250) in a 50-mL falcon tube at a ratio of 1:1 and added to another falcon tube containing Lymphoprep density gradient medium (Pancoll) (PAN BIOTECH, Aidenbach, Germany) in order to perform a density gradient centrifugation. The collected PBMC were washed twice with DPBS-EDTA. Erythrocytes were lysed and washed away with red blood cell (RBC) lysis buffer (Biolegend, San Diego, CA, USA) and another DPBS-EDTA washing step. The cells obtained were counted and plated at a density of 1–2 ×x 10⁶ cells/mL in a T-175 flask containing 40 mL of culture medium RPMI 1640 (PAN BIOTECH, Aidenbach, Germany) supplemented with 10% newborn calf serum (NCS) (Sigma, St. Louis, MO, USA penicillin and streptomycin P/S (Gibco, Gaithersburg, MD, USA), and 1 M Hepes (PAN BIOTECH, Aidenbach, Germany). After 2 h, the flask was changed in order to eliminate the adherent cells and 20 μl of IFN-gamma (ImmunoTools GmbH, Friesoythe, Germany) was added (2000 U μL⁻¹). On the next day, 100 μL of IL-1β (ImmunoTools GmbH, Friesoythe, Germany) (40 U μL⁻¹), 2 μL of anti-CD3 antibody (eBioscience, Thermo Fisher Scientific, Inc. San Diego, CA, USA) (1 mg/mL), as well as 24 μL of IL-2 (ImmunoTools GmbH, Friesoythe, Germany) (1000 U μL⁻¹) were added to the cells. Every 3 days, half of the medium was exchanged and 600 U/ mL IL-2 were added. After day 14 of culture, the cells were considered mature CIK cells.

### 4.2. Cell Lines and Cell Culture

Multiple myeloma cell line KMS-12 PE and U-266 (obtained from Leibniz Institute DSMZ Deutsche Sammlung von Mikroorganismen und Zellkulturen) were cultured in RPMI 1640 (PAN BIOTECH, Aidenbach, Germany) supplemented with 10% newborn calf serum (NCS) (Sigma, St. Louis, MO, USA) and 1% penicillin and streptomycin P/S (Gibco, Gaithersburg, MD, USA) at 37 °C in a humidified atmosphere with 5% CO_2_.

### 4.3. Cell Viability Analysis

Cell viability analysis was performed by a Cell Counting Kit-8 (CCK-8) assay. MM cell lines and CIK cells were exposed to different concentrations of pure cannabidiol (100%) (Santa Cruz Biotechnologie, Heidelberg, Germany) from 1 µM to 20 µM for 24 h at 37 °C. The effector cells (CIK cells) were co-cultured with target cells (KMS-12 PE) at effector and target (E∶T) ratios of 10∶1 and seeded into flat bottom 96-well plates. Next, 10 µL of CCK-8 reagent (Dojindo, Kumamoto, Japan) was added to the cells according to the manufacturer’s instructions. They were incubated for 1 h in the incubator; then, the absorbance was measured at 450 nm using a microplate reader. All the experiments were performed in triplicates, and the results were normalized. This experiment was replicated three times with CIK cells from three different donors.

### 4.4. LDH Assay

A commercial Pierce™ LDH Cytotoxicity Assay Kit (ThermoFisher, Waltham, MA, USA) was used according to the manufacturer’s instructions. MM cell lines and CIK cells were exposed to different concentrations of pure cannabidiol (Santa Cruz Biotechnologie, Heidelberg, Germany) from 1 µM to 20 µM for 24 h at 37 °C. The effector cells (CIK cells) were co-cultured with target cells (KMS-12 PE) at effector and target (E∶T) ratios of 10∶1 and seeded into 48-well plates. The absorption of the released LDH was then measured with a microplate reader at 490 nm and 680 nm. At the end of incubation, 25 µL of each sample was transferred to a 96-well flat bottom plate in different wells, and 25 µL of the reaction mixture was added to each well. To determine LDH activity, the 680 nm absorbance value is substracted from the 490 nm absorbance value. All experiments were performed in triplicates. Experiments were replicated three times with CIK cells from three different donors. In order to calculate % cytotoxicity, the following equation was applied to the corrected values:

$$\%\;\mathrm{citotoxicity}\;=\;\frac{\mathrm{Experimental}\;\mathrm{value}-\mathrm{Effector}\;\mathrm{Cells}\;\mathrm{Spontaneous}\;\mathrm{Control}-\mathrm{Target}\;\mathrm{Cells}\;\mathrm{Spontaneous}\;\mathrm{Control}}{\mathrm{Target}\;\mathrm{Cells}\;\mathrm{Maximum}\;\mathrm{Control}-\mathrm{Target}\;\mathrm{Cells}\;\mathrm{Spontaneous}\;\mathrm{Control}}\;\times \;100$$

To calculate % cytotoxicity of CBD on CIK cells or tumor cells, the following equation was applied to the corrected values:

$$\%\;\mathrm{citotoxicity}\;=\;\frac{\mathrm{Compound}-\mathrm{treated}\;\mathrm{LDH}\;\mathrm{activity}-\mathrm{Spontaneous}\;\mathrm{LDH}\;\mathrm{activity}}{\mathrm{Maximum}\;\mathrm{LDH}\;\mathrm{activity}-\mathrm{Spontaneous}\;\mathrm{LDH}\;\mathrm{activity}}\;\times \;100$$

### 4.5. Fluorescence-Activated Cell Sorting (FACS) Analysis

The following antihuman antibodies were used to stain cell surface markers to establish the CIK phenotype: CD3-fluorescein isothiocyanate (FITC) (Biolegend, San Diego, CA, USA), CD56-phycoerythrin (PE) (Biolegend, San Diego, CA, USA), CD4-allophycocyanin (APC) (Biolegend), CD8-Brilliant Violet 421(BV421) (Biolegend, San Diego, CA, USA), CD3-phycoerythrin (PE) (Biolegend, San Diego, CA, USA), CD56-allophycocyanin (APC) (Biolegend, San Diego, CA, USA), CD20-Pacific Blue (Biolegend, San Diego, CA, USA), and FITC-NKG2D (Biolegend, San Diego, CA, USA). For surface and intracellular CB2 receptor staining, CIK cells and tumor cells were fixed and permeabilized with Invitrogen Intracelluar Fix & Perm set kit (ThermoFisher, Waltham, MA, USA) according to the manufacturer’s instructions. The cells were stained with a FITC-conjugated antibody against CB2 (Cayman Chemical, Michigan, USA) and an anti-rabbit IgG FITC-conjugated anti-CB2 antibody (Cayman Chemical, Michigan, USA). Zombie Aqua™ Fixable Viability Kit (Biolegend, San Diego, CA, USA) permeant to cells with compromised membranes was used to stain the dead CIK cells and 7-Aminoactinomycin D (7-AAD) (Biolegend, San Diego, CA, USA) to stain the dead tumor cells. To assess the cytotoxicity of CIK cells in combination with pure cannabidiol (Santa Cruz Biotechnologie, Heidelberg, Germany) on multiple cell lines, the carboxyfluorescein succinimidyl ester (CFSE; ThermoFisher, Waltham, MA, USA)-labeled multiple myeloma cells were incubated along with Far Red (ThermoFisher, Waltham, MA, USA)-labeled CIK cells in an E:T ratio of 10∶1 and exposed to different concentrations of pure cannabidiol from 1 µM to 20 µM for 24 hours at 37 °C. Pure cannabidiol was first solved in DMSO and afterwards diluted within the corresponding RPMI medium (PAN BIOTECH, Aidenbach, Germany). The cell suspensions the were washed with DPBS (PAN BIOTECH, Aidenbach, Germany) twice. Finally, the dead cells were stained with Hoechst 33258 (Cayman Chemical, Michigan, USA) and Precision Count Beads™ (Biolegend, San Diego, CA, USA) were added.
